# Use of Nonpharmacologic Interventions by Adults With High-Impact Chronic Pain in the United States: A Cross-Sectional Analysis

**DOI:** 10.1155/prm/5213178

**Published:** 2025-04-07

**Authors:** Natasha L. Parman, Robert H. Schmicker, Sean D. Rundell

**Affiliations:** ^1^Department of Child, Family, and Population Health Nursing, University of Washington, Seattle, Washington, USA; ^2^Department of Biostatistics, University of Washington, Seattle, Washington, USA; ^3^Department of Rehabilitation Medicine, University of Washington, Seattle, Washington, USA; ^4^Department of Health Systems and Population Health, University of Washington, Seattle, Washington, USA

## Abstract

**Introduction:** Few studies compare differences in the use of nonpharmacologic interventions (NPIs) between those with high-impact chronic pain (HICP) and low-impact chronic pain (LICP) or describe differences in the use of NPIs by locations of bothersome pain.

**Objectives:** To describe the use of NPIs in HICP and LICP subgroups and to examine the association between locations of bothersome pain and use of NPIs among those with HICP.

**Methods:** We used data from the 2019 National Health Interview Survey. After identifying respondents who reported having chronic pain, we then created high and low pain impact subgroups. Additional variables in our analyses included sociodemographic data, health characteristics, and pain management characteristics. Our analysis included descriptive statistics, Chi-squared tests, and adjusted survey-weighted logistic regression models.

**Results:** The estimated prevalence of chronic pain in US adults was 19.9% (95% CI: 19.5–20.0), with 36.4% (95% CI: 35.1–38.0) of that group having HICP. Of those with HICP, 69.7% (95% CI: 67.6–71.9) reported using ≥ 1 NPIs in the past 3 months, compared to 62.9% (95% CI: 61.1–64.6) with LICP. The most frequently used NPI was physical, rehabilitative, or occupational therapy (25.9%, 95% CI: 24.0–27.9), and the least used was a peer support group (2.7%, 95% CI: 2.0–3.6). Among those with HICP, bothersome back pain (OR = 1.52, 95% CI: 1.19–1.95) and upper extremity pain (OR = 1.26, 95% CI: 1.003–1.59) are associated with the greater use of any NPIs compared to those without bothersome pain at these sites, respectively.

**Conclusion:** Our findings highlight that most US adults with HICP have recently used NPIs to manage their pain, but the use of specific NPIs varied considerably. The odds of using NPIs were different depending on the locations of bothersome pain. Future work should examine barriers for access to specific NPIs or the use of NPIs by locations of bothersome pain.

## 1. Introduction

Chronic pain is a complex health condition affecting an estimated 51.6 million (20.9%) US adults in 2021 with numerous potential sources and various contributing factors [1]. There are well-known links between chronic pain and other chronic health conditions, as well as increased mortality [2, 3]. Chronic pain negatively contributes to interference with daily activities and work, and it is a significant negative economic burden on society [4, 5].

There is variability in how much chronic pain impacts individuals' daily activities and work [6]. The 2016 National Pain Strategy advocated for more precise estimates of chronic pain in the US population, including pain impact, and they recommended identifying high-impact chronic pain (HICP)—defined as chronic pain “that is associated with substantial restriction of participation in work, social, and self-care activities.” [6] In response to this call, researchers have begun to estimate the prevalence of HICP, with Rikard et al. estimating that 17.1 million (6.9%) of US adults experienced HICP in 2021, and to specifically study this important subgroup. The Centers for Disease Control and Prevention (CDC) recommends a multimodal approach to treating chronic pain, with nonpharmacologic interventions (NPIs) as first-line treatments [7]. Previous studies have examined the use of NPIs for the treatment of chronic pain and identified that these interventions are underutilized by those with any chronic pain and HICP, and use varies based on sociodemographic, health, and pain condition characteristics, such as age, sex, income, education, geographic location, and the presence of a disability [8–15].

However, to our knowledge, none have described the differences in the use of different types of NPIs between HICP and low-impact chronic pain (LICP) or examined the association between locations of bothersome pain and use of NPIs in adults with HICP. These differences are important to understand, as they can inform stakeholders where certain treatments may be under or overutilized, may guide healthcare policy development to improve the use of NPIs specifically for HICP, and may bring awareness to treatment inequities.

To address these gaps, our objectives are (1) to describe the use of NPIs in HICP and LICP subgroups and (2) to examine the association between locations of bothersome pain and use of NPIs among those with HICP, controlling for numerous sociodemographic and health covariates. By describing the association between locations of bothersome pain and use of NPIs, research and policy efforts can be more precisely targeted to better understand usage differences and potentially improve access to care.

## 2. Materials and Methods

### 2.1. Study Design and Data Source

This is a cross-sectional study using the public-use data files of the 2019 National Health Interview Survey (NHIS) [16]. The NHIS is a cross-sectional household survey targeting the civilian noninstitutionalized population in the United States (US), and Duca et al. consider it to be the best single source for pain surveillance [17]. The survey is conducted in-person with computer assistance and may include telephone interviewing as needed. Geographically clustered sampling techniques are used so the sample is nationally representative when sampling weights are used. This study followed the Strengthening the Reporting of Observational Studies in Epidemiology (STROBE) guidelines for cohort studies [18].

### 2.2. Ethics Statement

Data collected for the 2019 NHIS were approved by the National Center for Health Statistics (NCHS) Research Ethics Review Board. All respondents provide verbally informed consent. The dataset is deidentified. As defined by federal regulations and guidance, this dataset does not involve “human subjects research,” and therefore, it is not required to apply for the Institutional Review Board review nor an exempt determination.

### 2.3. Study Participants

We identified adult (age 18 and older) participants with chronic pain by their answer to the following question: “In the past three months, how often did you have pain?” If the participant answered either “every day” or “most days,” they were considered to have chronic pain [1]. Other potential responses included “some days” or “never.” The sample was further stratified by the participants' pain impact, where respondents were asked “Over the past 3 months, how often did your pain limit your life or work activities?” The participant was considered to have HICP if they answered “every day” or “most days,” and LICP if they answered “some days” or “never” [6].

### 2.4. Sociodemographic Data

Sociodemographic data used in our analyses included age, sex, race and ethnicity, marital status, sexual orientation, veteran status, nativity, highest education level, income relative to the Federal Poverty Level (FPL), employment status, insurance coverage for those under 65 years old, insurance coverage for those 65 years of age and older, and urbanicity.

Some variables were recategorized by the authors for the ease of interpretation. This included age, education level, income relative to the FPL, and employment status. We recategorized age into the following levels: 18–24, 25–44, 45–64, 65–84, and 85+. Education was recategorized into the following levels: no high school diploma, high school graduate or passed general educational development (GED) test, some college, or completed a Bachelor's degree or higher. Income relative to the FPL was recategorized into the following levels: < 100% of FPL, 100% to < 200% FPL, 200% to < 400% FPL, and ≥ 400% FPL. Employment was recategorized into the following levels: employed full time, retired, unemployed, or other. The levels at which variables were recategorized were determined *a priori*.

Other variables were not recategorized by the authors, including race and ethnicity, marital status, sexual orientation, veteran status, nativity, urbanicity, and insurance coverage. Urbanicity was determined using the 2013 NCHS Urban–Rural Classification Scheme for Counties [19].

### 2.5. Health Variables

Health characteristics included self-reported height and weight. Using height and weight, we calculated body mass index and categorized it as underweight (< 18.5 kg/m²), normal (18.5–24.9 kg/m²), overweight (25.0–29.9 kg/m²), and obese (≥ 30.0 kg/m²) [20]. General health status was self-rated as excellent, very good, good, fair, or poor. Respondents were asked if they had ever been diagnosed with the following chronic conditions: hypertension, high cholesterol, coronary heart disease, stroke, dementia, cancer, diabetes, arthritis, chronic obstructive pulmonary disorder, asthma, anxiety, or depression. We determined the number of chronic conditions by summing the number of individual comorbidities and categorized them as zero, one, two, three, or four or more conditions.

We used data regarding pain amount and pain location. Pain amount was captured by the following question: “Thinking about the last time you had pain, how much pain did you have?” and was not specific to a body region. Possible responses included a little, a lot, somewhere in between a little and a lot, or no answer provided. Pain locations and bothersomeness of that specific pain area were also captured. As an example for back pain, the survey asked, “Over the past 3 months, how much have you been bothered by back pain? Would you say not at all, a little, a lot, or somewhere in between?” This was repeated for the hands, arms, or shoulders; hips, knees, or feet; headache or migraine; abdomen, pelvis, or genitals; and toothache or jaw. Participants were considered to have pain at a location if they answered at least “a little” for the bothersomeness at that site.

### 2.6. Pain Management Variables

Pain management strategies were identified by participants' responses to questions on whether or not they had used the following NPIs in the past 3 months to manage their pain: physical, occupational, or rehabilitative therapy; spinal manipulation or other forms of chiropractic care; talk therapies such as cognitive–behavioral therapy; a chronic pain self-management program or workshop; chronic pain peer support groups; Yoga or Tai Chi; massage; meditation, guided imagery, or other relaxation techniques; and other methods. We categorized the number of NPIs used as zero, one, or two or more. Regarding medication management, we used data on self-reported prescription opioid use, specifically for chronic pain, in the past 3 months. The amount or type of opioid was not collected. Data regarding the use of non-opioid pain relievers were not collected in the 2019 NHIS.

### 2.7. Statistical Methods

Descriptive statistics are presented in tables with weighted percentages, 95% confidence intervals, and Chi-Square *p*-values. Population-based estimates were calculated using the sampling weights provided by the NCHS [21]. If a respondent refused, their answer was not ascertained, or they did not know the answer, these were recoded as “no answer” when appropriate. We described the use of each type of NPI stratified by pain impact, locations of bothersome pain, and use of opioids. Survey design-weighted logistic regression models examined the association between using at least one NPI and bothersome pain location among those with HICP. Models were adjusted for sex, age, race, marital status, income relative to the FPL, employment status, education status, insurance status, body mass index, number of comorbidities, and urbanicity. We also calculated how many respondents used opioids and zero NPIs, opioids and one NPI, and opioids and two or more NPIs.

For secondary analyses, similar models as above were used with each specific type of NPI as the outcome. In addition, we included the number of total locations of bothersome pain as a count variable instead of each pain site individually. Because missing data were small in frequency (< 1%) and thought to be missing completely at random, imputation methods were not used. Because the analysis was descriptive and exploratory in nature, adjustment for multiple comparisons was not used. Analyses were performed with R (v 4.1.1) using the svydesign function as part of the survey package [22, 23].

## 3. Results

### 3.1. Participants

There were 31 997 adult NHIS participants in 2019, and 7184 (22.5%) reported having chronic pain, representing a weighted population of 51 103 668 individuals. Of those, 2666 (37.1%) had HICP, representing a weighted population of 18 250 510 individuals, and 4508 had LICP, representing 31 853 158 individuals. Population-based estimates for the HICP subgroup show that 59.0% (95% CI: 56.6%–61.3%) were female, 45.6% (95% CI: 43.3%–47.9%) were 45–64 years old, 23.0% (95% CI: 21.0%–25.0%) reported an income < 100% of the FPL, and 27.7% (95% CI: 25.6%–29.9%) were employed full time. We found 24% (95% CI: 22.0%–26.1%) of participants reported their health status as “Poor,” and 49.1% (95% CI: 46.8%–51.4%) reported having four or more chronic conditions. The most common category of chronic condition, reported by 63.4% (95% CI: 61.1%–65.7%) of participants, was “some form of arthritis, rheumatoid arthritis, gout, lupus, or fibromyalgia.” There were significant differences between the HICP and LICP groups in terms of age, sex, marital status, education level, income, employment, insurance, self-rated general health, multimorbidity, and the presence of all individual comorbidities.  Table 1 presents all sociodemographic and health data for both impact subgroups.

### 3.2. Use of NPIs and Bothersome Pain Locations

In the HICP subgroup, 30.3% (95% CI: 28.1%-32.4%) reported no use of NPIs in the past 3 months, 33.7% (95% CI: 31.5%–36.0%) reported using one NPI, and 36.0% (95% CI: 33.9%–38.3%) reported using two or more NPIs. The most used NPIs were physical, occupational, or rehabilitative therapy (25.9%, 95% CI: 24.0%–27.9%), massage (18.2%, 95% CI: 16.4%–20.1%), and meditation, guided imagery, or other forms of relaxation techniques (17.8%, 95% CI: 16.2%–19.6%). Prescription opioid use for the management of chronic pain was reported by 28.6% (95% CI: 26.4%–30.9%) of those with HICP, and a majority of opioid users also reported the use of NPIs. Compared to the LICP subgroup, the HICP subgroup had a greater use of NPIs that include cognitive, emotional, social, and rehabilitative interventions (e.g., physical therapy, talk therapy, and peer support) and similar use of NPIs that are traditionally considered complementary to conventional care (massage and spinal manipulation or other chiropractic care) (Table 2).

Regarding locations of bothersome pain, the most common locations where participants with HICP reported “at least a little” pain were the hips, knees, or feet (85.4%, 95% CI: 83.6%–87.0%) and the back (84.4%, 95% CI: 82.7%–85.9%). Regardless of location, 57.6% (95% CI: 55.3%–59.9%) reported their pain bothersomeness as “a lot” and 36.6% (95% CI: 34.4%–38.9%) reported their pain bothersomeness as “somewhere in between a little and a lot.” Compared to those with LICP, the HICP subgroup reported having a much greater presence of highly bothersome back pain, upper extremity pain, and lower extremity pain.  Table 3 describes the bothersomeness amount and locations for both pain impact groups.

Among those with HICP, back pain (OR = 1.52, 95% CI: 1.19–1.95) and hand, arm, or shoulder pain (OR = 1.26, 95% CI: 1.003–1.59) were associated with the greater use of any NPI compared to those with HICP and no pain at these sites, respectively. Those with back pain had higher odds of using spinal manipulation or other forms of chiropractic care (OR = 2.20, 95% CI: 1.49–3.25), massage (OR = 1.51, 95% CI: 1.10–2.09), a chronic pain self-management program or workshop (OR = 1.98, 95% CI: 1.28–3.06), and physical, occupational, or rehabilitative therapy (OR = 1.46, 95% CI: 1.13–1.88) compared to those without back pain. Those with a toothache or jaw pain had increased odds of using group/peer (OR = 2.24, 95% CI: 1.10–4.56), meditation, guided imagery, or other forms of relaxation techniques (OR = 1.82, 95% CI: 1.26–2.61), or talk therapy (OR = 1.80, 95% CI: 1.04–3.11) versus those without a toothache or jaw pain. Full results are available in Table 4.

We found that the odds of using any NPI increased by 11% for every additional bothersome pain site present (OR = 1.11, 95% CI: 1.01–1.21) among those with HICP. A greater number of bothersome pain sites was associated with the increased use of the following specific NPIs: massage (OR = 1.29, 95% CI: 1.17–1.43), talk therapy (OR = 1.29, 95% CI: 1.05–1.60), meditation, guided imagery, or other forms of relaxation techniques (OR = 1.25, 95% CI: 1.12–1.39), Yoga or Tai Chi (OR = 1.24, 95% CI: 1.02–1.51), and spinal manipulation or other forms of chiropractic care (OR = 1.18, 95% CI: 1.04–1.33) (Table 5).

## 4. Discussion

This study described the use of NPIs among US adults with HICP and LICP in a nationally representative sample. Further, we examined the association between locations of bothersome pain and odds of using NPIs for those with HICP, controlling for several important covariates. Approximately 70% of those with HICP reported the recent use of any NPI, and the HICP group used most types of NPIs more frequently except spinal manipulation or other chiropractic care, massage, Yoga or Tai Chi, and “other” interventions compared to those with LICP. Locations of bothersome pain were also associated with the NPI use. We found increased odds of using one or more NPIs for those with bothersome back pain and pain of the upper extremity, and those with bothersome back pain and upper extremity pain used most individual NPIs more frequently than those with HICP but no bothersome back pain or upper extremity pain, respectively.

Survey research can provide a population-level perspective on how chronic pain is managed in health systems and the community. The NHIS provides timely and nationally representative information regarding illness, disability, and care received for those conditions in the US [17]. Important trends can be identified over time, and the data can be used to track progress toward national health objectives [16]. The data can help stakeholders identify where healthcare use may be appropriately utilized, overutilized, or underutilized for certain medical conditions or where treatment inequities exist among those with specific demographic and socioeconomic characteristics.

As noted earlier, most respondents indicated the use of some NPI in the past 3 months to manage their HICP. This amount of use seems appropriate given that some type of ongoing pain management may be needed for many people with a chronic condition affecting their function. It also seems appropriate that NPIs are much more commonly used than opioids for managing chronic pain. It is possible that more than 70% of people with HICP have some experience with NPIs, though. Those with HICP who did not use an NPI in the past 3 months may have used NPIs in the more distant past, may cope with their pain without using NPIs, or have found using NPIs ineffective and have chosen to no longer use them. These findings provide insight into what types of NPIs people with HICP are using, but we do not know whether some NPIs were practiced with guidance or independently, such as Yoga and Tai Chi or meditation. It may be helpful for future research to investigate how these interventions were practiced, as there may be important and informative differences in the rates of guided versus independent practice. Lastly, our work shows that it is more common for people with HICP to use opioids with NPIs than without NPIs. We consider this appropriate, as guidelines recommend using both approaches together when needed for effective management [7].

Our results show that a few US adults with HICP used talked therapy, a self-management program for pain, or a chronic pain peer support group. While it is not surprising these interventions were used less commonly compared to the greater rates of physical therapy, occupational therapy, or rehabilitative therapy, spinal manipulation or other forms of chiropractic care, and massage, this likely demonstrates the underuse of self-management strategies, psychological therapies, and peer support, given the evidence to support the benefit of self-management, psychological therapies, and peer support in HICP [24–26]. This low amount of use may reflect a lack of access to these interventions since their availability is often limited [27]. There may also be cultural beliefs that physical pain needs body-focused approaches more than or in contrast to psychosocial approaches, which may affect care-seeking behaviors [28, 29].

Our study uses NHIS data to better understand chronic pain management in the US by examining the NPI use stratified by pain impact, as well as examining variation of use across the location of bothersome pain and number of bothersome pain locations. This adds to the current literature regarding management of chronic pain.

Groenewald et al. used NHIS data from 2019 to describe the use of NPIs by US adults with chronic pain, but they did not stratify their results by pain impact [8]. They also reported that the most used NPIs for chronic pain were physical, occupational, or rehabilitative therapy (estimated 18.8% (9.4 million) adults), followed by massage (estimated 17.6% (8.8 million) adults), meditation, guided imagery, or other relaxation techniques (estimated 15.6% (7.8 million) adults). This aligns with the top three most used NPIs for our HICP subgroup only. Even though the LICP subgroup had the same top three NPIs, the ranking was in a different order: massage; meditation, guided imagery, or other relaxation techniques; and then physical therapy, occupational therapy, or rehabilitative therapy.

Rodgers-Melnick et al. also examined the use of NPIs in US adults with chronic pain using the same 2019 NHIS data, but they focused on nonpharmacologic integrative health and medicine (IHM) modalities only: spinal manipulation or other forms of chiropractic care, yoga or Tai Chi, massage, or meditation, guided imagery, or other relaxation techniques [13]. They looked at the use of any of these interventions collectively and did not report engagement in each respective modality. They investigated numerous predictors of using these modalities, including pain-related predictors, and found that the number of pain locations was significantly different between those engaging in these modalities compared to those not engaging in integrative modalities, like our study. They did not include individual pain locations as a predictor. They also found that those who engaged with ≥ 1 integrative modality reported a higher prevalence of pain, which limited life or work activities. While a direct comparison cannot be made, as we included conventional interventions (physical therapy, occupational therapy, or rehabilitative therapy, talk therapies, self-management programs, and chronic pain peer support groups) in our analyses, our results also show more engagement in NPIs among people with higher impact pain. This suggests that people with a greater need for treatment, those with HICP, seek conventional and integrative NPIs.

Beyond examining pain management strategies, our findings corroborate that the prevalence of chronic pain and HICP has been relatively stable between 2016 and 2021. The Centers for Disease Control and Prevention (CDC) analyzed data from the 2016 NHIS and reported that 20.4% and 8.0% of US adults had chronic pain and HICP, respectively [30]. Rikard et al. used data from the 2019–2021 NHIS and reported that 20.9% and 6.9% of US adults had chronic pain and HICP, respectively, in 2021 [1]. Using the same definition of chronic pain and HICP as Dahlhamer and Rikard, we estimated that 20.0% and 7.3% US adults had chronic pain and HICP in 2019, respectively. 

Our study has two important strengths to highlight. By using NHIS data and sample weighing, we provided population-level estimates that are nationally representative of the US adult population. Given the large sample size, we also have a degree of precision in our estimates that is not possible with smaller samples. We add new population-based information on how pain location is associated with NPI use.

There are a few important limitations to note when using survey data. Data collected by self-report may be influenced by recall bias [17] and social desirability bias. However, with a 3-month recall for the use of NPIs, recall bias is likely minimized [31]. Our models were adjusted for several important covariates, but there may have been other variables we did not adjust for that could account for some residual confounding for the use of NPIs (e.g., veteran status and sexual orientation).

Specific to the 2019 NHIS survey, some commonly used NPIs were not included. For example, potentially common interventions such as acupuncture, devices such as electrical stimulation devices, and nutritional approaches were omitted. The use of NPIs may not be meaningfully underestimated since there was an “Other” option to address this, and this category of NPI was the most frequently reported (see Table 2). Thus, there remains gaps in our understanding of what NPIs people with chronic pain use and may prefer. Future research should explore what other NPIs are used. Perceived effectiveness in managing one's pain is included in the interview; however, we did not report on these data because it is not specific to the use of NPIs. We did include data on opioid use for pain management, but we cannot report on more granular data such as dose or frequency. We could not examine the use of non-opioid pain medication in conjunction with NPIs. Another limitation of using the 2019 NHIS data is that the interview does not specifically ask about neck pain. It is possible that some respondents with chronic neck pain were not captured, and we cannot investigate if chronic neck pain is associated with greater or lesser use of NPIs. Lastly, these data were collected before the start of the COVID-19 pandemic, and it is possible that the use of NPIs for HICP or LICP has changed since the pandemic, including how they are delivered (e.g., telehealth). Other authors have examined this in certain cohorts [9], and we expect that this will be a relevant line of research in the future.

## 5. Conclusion

We found that most US adults with HICP are using NPIs to manage their pain. However, those with bothersome back pain and upper extremity pain have the more commonly used NPIs compared to those without bothersome pain at those sites, even when adjusting for several important covariates. This builds on previous knowledge that has shown differences in care based on pain-related factors by suggesting that bothersome pain location is associated with the use of NPIs for chronic pain.

## Figures and Tables

**Table 1:** Respondent characteristics by impact level (weighted % and weighted 95% CI).

	Low impact	High impact	
Unweighted population	4508	2666	

Weighted population	31,853,158	18,250,510	*p* value^2^

*Age*			∗∗∗
18–24	5.2 (4.3–6.3)	3.3 (2.4–4.6)	
25–44	24.4 (22.9–26.1)	17.7 (15.9–19.7)	
45–64	39.6 (37.8–41.3)	45.6 (43.3–47.9)	
65–84	27.6 (26.2–29.1)	28.3 (26.4–30.3)	
> 85	3.1 (2.7–3.7)	4.9 (4.0–6.1)	

*Sex*			∗∗∗
Male	47.3 (45.6–49.1)	41.0 (38.7–43.4)	
Female	52.6 (50.9–54.4)	59.0 (56.6–61.3)	
Unknown	0.0 (0.0–0.2)	0.0 (0.0–0.0)	

*Race/ethnicity*			
White, non-Hispanic	74.1 (72.5–75.7)	71.6 (69.3–73.7)	
Black, non-Hispanic	10.6 (9.6–11.8)	11.6 (10.2–13.2)	
Asian, non-Hispanic	2.1 (1.6–2.8)	1.7 (1.1–2.5)	
AI/AN, non-Hispanic	2.1 (1.7–2.6)	2.4 (1.8–3.2)	
Other, non-Hispanic	1.2 (0.9–1.8)	1.0 (0.5–1.7)	
Hispanic	9.8 (8.7–11.0)	11.7 (10.2–13.5)	

*Marital status*			∗∗∗
Married	53.3 (51.5–55.0)	46.3 (44.0–48.7)	
Divorced	16.4 (15.2–17.7)	20.0 (18.4–21.8)	
Separated	1.7 (1.3–2.2)	2.2 (1.7–3.0)	
Never married	18.8 (17.4–20.4)	16.8 (15.0–18.8)	
Widowed	9.3 (8.4–10.2)	13.3 (11.9–14.8)	
No answer^1^	0.5 (0.3–0.8)	1.3 (0.8–2.2)	

*Sexual orientation*			
Straight	94.6 (93.7–95.3)	93.8 (92.4–95.0)	
Gay	1.1 (0.8–1.5)	1.2 (0.8–1.7)	
Bisexual	2.1 (1.7–2.7)	1.5 (1.0–2.2)	
Something else	0.4 (0.2–0.7)	0.6 (0.3–1.1)	
No answer^1^	1.8 (1.3–2.3)	2.9 (2.1–4.1)	

*Veteran status*			
Yes	12.3 (11.2–13.4)	13.2 (11.7–14.7)	
No	87.4 (86.3–88.5)	85.8 (84.1–87.3)	
No answer^1^	0.3 (0.1–0.5)	1.1 (0.6–1.8)	

*Nativity*			
Yes	89.7 (88.5–90.9)	87.9 (86.1–89.5)	
No	10.0 (8.9–11.2)	11.0 (9.5–12.7)	
No answer^1^	0.3 (0.2–0.5)	1.1 (0.6–1.8)	
Height (inches), mean (sd)	69.2 (0.2)	69.3 (0.2)	
Weight (pounds), mean (sd)	262.0 (4.4)	283.2 (6.8)	∗∗
Body mass index, mean (sd)	29.1 (0.1)	29.8 (0.2)	∗∗
Underweight	0.4 (0.2–0.7)	0.9 (0.6–1.4)	
Normal	24.5 (23.0–26.2)	23.0 (21.1–25.1)	
Overweight	35.3 (33.5–37.1)	32.0 (29.8–34.4)	
Obese	39.7 (37.9–41.6)	44.0 (41.6–46.5)	

*Highest education level*			∗∗∗
No high school or GED	13.3 (12.0–14.7)	19.4 (17.5–21.5)	
High school or GED	29.2 (27.5–30.8)	31.4 (29.2–33.6)	
Some college	34.0 (32.4–35.7)	32.8 (30.7–35.1)	
Bachelor or higher	22.7 (21.4–24.1)	15.6 (14.1–17.1)	
No answer^1^	0.8 (0.5–1.3)	0.8 (0.4–1.5)	

*Income relative to FPL*			∗∗∗
< 100%	12.2 (11.0–13.4)	23.0 (21.0–25.0)	
100%–200%	20.1 (18.7–21.6)	27.4 (25.4–29.6)	
200%–400%	32.0 (30.4–33.7)	28.9 (26.8–31.1)	
> 400%	35.7 (34.1–37.4)	20.7 (18.9–22.5)	

*Employment status*			∗∗∗
Full time	55.9 (54.1–57.6)	27.7 (25.6–29.9)	
Retired	1.9 (1.4–2.5)	1.4 (0.9–2.2)	
Unemployed	26.3 (24.9–27.7)	28.1 (26.2–30.1)	
Other	15.6 (14.3–17.1)	41.7 (39.4–44.1)	

*Insurance*			
*n* < 65 years	2728	1573	∗∗∗
Private	62.1 (59.9–64.3)	42.2 (39.3–45.2)	
Medicaid	15.6 (14.0–17.4)	30.5 (27.7–33.3)	
Other	7.2 (6.2–8.4)	14.6 (12.6–16.8)	
Uninsured	14.7 (13.1–16.5)	12.7 (10.7–15.0)	

*n* ≥ 65 years	1776	1090	∗∗∗
Private	41.0 (38.3–43.7)	33.4 (30.1–36.8)	
Dual	7.6 (6.2–9.3)	13.8 (11.4–16.6)	
Medicare advantage	28.9 (26.5–31.4)	27.5 (24.4–30.8)	
Medicare	11.7 (9.9–13.7)	11.9 (9.9–14.4)	
Other	9.6 (8.1–11.3)	12.1 (9.8– 4.9)	

*Urbanicity*			
Large central metropolitan	24.2 (22.7–25.8)	25.0 (23.0–27.1)	
Large fringe metropolitan	22.0 (20.6–23.5)	21.1 (19.1–23.2)	
Medium and small metropolitans	34.6 (32.9–36.3)	32.8 (30.7–35.0)	
Nonmetropolitan	19.2 (17.8–20.6)	21.1 (19.3–23.0)	

*Self-rated general health*			∗∗∗
Excellent	9.3 (8.3–10.3)	3.5 (2.7–4.4)	
Very good	27.4 (25.8–29.0)	11.9 (10.5–13.5)	
Good	37.0 (35.3–38.7)	25.3 (23.3–27.3)	
Fair	21.1 (19.6–22.6)	35.3 (33.0–37.5)	
Poor	5.3 (4.5–6.2)	24.0 (22.0–26.1)	

*Total medical conditions*			∗∗∗
0	15.2 (13.9–16.6)	6.4 (5.3–7.8)	
1	17.6 (16.3–19.0)	10.6 (9.2–12.2)	
2	20.2 (18.8–21.7)	15.0 (13.4–16.8)	
3	18.4 (17.1–19.8)	18.8 (17.0–20.7)	
4+	28.6 (27.1–30.2)	49.1 (46.8–51.4)	

*Individual medical conditions*			
Hypertension	46.4 (44.7–48.2)	58.2 (55.8–60.5)	∗∗∗
High cholesterol	37.9 (36.3–39.7)	45.1 (42.8–47.4)	∗∗∗
Coronary heart disease	8.6 (7.7–9.6)	13.0 (11.6–14.6)	∗∗∗
Stroke	5.4 (4.7–6.2)	10.0 (8.6–11.6)	∗∗∗
Dementia	1.1 (0.8–1.5)	4.0 (3.1–5.1)	∗∗∗
Cancer	15.2 (14.0–16.4)	17.9 (16.2–19.7)	∗
Diabetes	15.5 (14.3–16.8)	21.9 (20.1–23.8)	∗∗∗
Arthritis	48.1 (46.3–49.9)	63.4 (61.1–65.7)	∗∗∗
Chronic obstructive pulmonary disorder	8.2 (7.3–9.2)	19.9 (18.2–21.7)	∗∗∗
Asthma	18.7 (17.3–20.1)	24.5 (22.5–26.6)	∗∗∗
Anxiety	23.2 (21.7–24.7)	39.3 (37.0–41.6)	∗∗∗
Depression	26.0 (24.5–27.5)	46.3 (43.9–48.6)	∗∗∗

*Note: n* (%) represents sample frequency and weight survey percentages; mean (sd) represents weighted survey means and standard deviations.

Abbreviations: AI/AN, American Indian or Alaska Native; CI, confidence interval; FPL, federal poverty level; GED, general education diploma.

^∗^Significant at *p* < 0.05.

^∗∗^Significant at *p* < 0.01.

^∗∗∗^Significant at *p* < 0.001.

^1^No answer includes three responses—“refused,” “not ascertained,” and “don't know.”

^2^ *p* value key.

**Table 2:** Nonpharmacologic intervention use for chronic pain by impact level (weighted % and weighted 95% CI).

	Low impact	High impact	
Unweighted population	4508	2666	

Weighted population	31,853,158	18,250,510	*p* value^1^

*Nonpharmacologic intervention*			∗∗∗
0 total	37.1 (35.4–38.9)	30.3 (28.1–32.4)	
1 total	34.1 (32.5–35.8)	33.7 (31.5–36.0)	
≥ 2 total	28.7 (27.2–30.4)	36.0 (33.9–38.3)	
Physical, rehabilitative, or occupational therapy	14.8 (13.6–16.1)	25.9 (24.0–27.9)	∗∗∗
Spinal manipulation or other chiropractic care	11.4 (10.4–12.6)	12.0 (10.6–13.6)	
Talk therapies	2.8 (2.3–3.4)	5.7 (4.7–6.8)	∗∗∗
Self-management program for pain	3.3 (2.7–4.0)	8.4 (7.2–9.8)	∗∗∗
Chronic pain peer support group	1.3 (1.0–1.9)	2.7 (2.0–3.6)	∗∗
Yoga or Tai Chi	9.3 (8.3–10.3)	7.3 (6.1–8.8)	∗
Massage	17.2 (15.9–18.6)	18.2 (16.4–20.1)	
Meditation, guided imagery, or other relaxation	14.3 (13.1–15.6)	17.8 (16.2–19.6)	∗∗
Other methods for pain	39.4 (37.7–41.1)	38.6 (36.3–40.9)	

*Prescription opioid use past 3 months for chronic pain*			∗∗∗
No	89.7 (88.5–90.8)	71.4 (69.1–73.6)	
Yes	10.3 (9.2–11.5)	28.6 (26.4–30.9)	
And no NPI	3.7 (3.0–4.5)	7.3 (6.1–8.7)	
And 1 NPI	3.5 (2.9–4.3)	10.5 (9.0–12.3)	
And ≥ 2 NPIs	3.1 (2.6–3.8)	10.8 (9.5–12.3)	

Abbreviations: CI, confidence interval; NPI, nonpharmacologic intervention.

^∗^Significant at *p* < 0.05.

^∗∗^Significant at *p* < 0.01.

^∗∗∗^Significant at *p* < 0.001.

^1^ *p* value key.

**Table 3:** Pain location and intensity by impact level (weighted % and weighted 95% CI).

	Low impact	High impact	
Unweighted population	4508	2666	

Weighted population	31,853,158	18,250,510	*p* value^2^

*Pain amount*			∗∗∗
A little	24.1 (22.6–25.6)	5.6 (4.6–6.8)	
Between a little and a lot	55.1 (53.3–56.9)	36.6 (34.4–38.9)	
A lot	20.8 (19.3–22.3)	57.6 (55.3–59.9)	
No answer^1^	0.0 (0.0–0.1)	0.2 (0.1–0.5)	

*Back pain*			∗∗∗
Not at all	23.7 (22.2–25.2)	15.4 (13.9–17.1)	
At least a little	76.2 (74.6–77.6)	84.4 (82.7–85.9)	
A little	26.0 (24.5–27.6)	15.9 (14.3–17.6)	
Between a little and a lot	17.8 (16.5–19.2)	12.9 (11.3–14.7)	
A lot	32.4 (30.7–34.1)	55.6 (53.2–57.9)	
No answer^1^	0.2 (0.1–0.4)	0.2 (0.1–0.5)	

*Pain in hands, arms, or shoulders*			∗∗∗
Not at all	35.1 (33.4–36.8)	26.4 (24.4–28.5)	
At least a little	64.8 (63.1–66.5)	73.2 (71.0–75.2)	
A little	24.9 (23.4–26.4)	17.5 (15.9–19.3)	
Between a little and a lot	15.4 (14.2–16.7)	11.5 (10.1–13.1)	
A lot	24.5 (23.0–26.0)	44.1 (41.8–46.4)	
No answer^1^	0.1 (0.1–0.3)	0.4 (0.2–0.9)	

*Pain in hips, knees, or feet*			∗∗∗
Not at all	24.9 (23.4–26.4)	14.2 (12.6–16.0)	
At least a little	75.0 (73.4–76.5)	85.4 (83.6–87.0)	
A little	22.9 (21.4–24.4)	14.2 (12.6–15.9)	
Between a little and a lot	17.7 (16.4–19.1)	10.5 (9.0–12.2)	
A lot	34.4 (32.7–36.1)	60.7 (58.4–63.0)	
No answer^1^	0.1 (0.1–0.3)	0.4 (0.2–0.8)	

*Headache or migraine*			∗∗∗
Not at all	61.4 (59.6–63.1)	51.7 (49.3–54.0)	
At least a little	38.4 (36.6–40.2)	47.8 (45.5–50.2)	
A little	20.5 (19.1–22.1)	20.8 (19.0–22.8)	
Between a little and a lot	7.2 (6.3–8.3)	8.5 (7.1–10.0)	
A lot	10.7 (9.6–11.8)	18.5 (16.7–20.5)	
No answer^1^	0.2 (0.1–0.4)	0.5 (0.2–0.9)	

*Abdominal, pelvic, or genital pain*			∗∗∗
Not at all	81.0 (79.5–82.3)	70.1 (68.0–72.2)	
At least a little	18.8 (17.4–20.2)	29.4 (27.3–31.5)	
A little	8.9 (8.0–10.0)	11.7 (10.3–13.2)	
Between a little and a lot	4.0 (3.3–4.9)	5.4 (4.4–6.5)	
A lot	5.8 (5.0–6.7)	12.3 (10.9–14.0)	
No answer^1^	0.2 (0.1–0.5)	0.5 (0.3–0.9)	

*Toothache or jaw pain*			∗∗∗
Not at all	81.7 (80.2–83.0)	74.8 (72.7–76.8)	
At least a little	18.2 (16.8–19.7)	24.7 (22.7–26.7)	
A little	9.9 (8.9–11.0)	13.4 (11.8–15.2)	
Between a little and a lot	2.7 (2.1–3.3)	3.4 (2.7–4.4)	
A lot	5.7 (4.8–6.7)	7.8 (6.7–9.1)	
No answer^1^	0.1 (0.0–0.3)	0.5 (0.3–1.0)	

*Note: n* (%) represents sample frequency and weight survey percentages.

Abbreviation: CI, confidence interval.

^∗^Significant at *p* < 0.05.

^∗∗^Significant at *p* < 0.01.

^∗∗∗^Significant at *p* < 0.001.

^1^No answer includes three responses—“refused,” “not ascertained,” and “don't know.”

^2^ *p* value key.

**Table 4:** Adjusted logistic regression estimates for NPI use outcomes, OR (95% CI), in relation with bothersome pain sites for patients with high-impact chronic pain.

	Back pain	Hands, arms, or shoulder pain	Hips, knees, or feet pain	Headache or migraine	Abdominal, pelvic, or genital pain	Toothache or jaw pain
NPI ≥ 1	**1.52 (1.19**–**1.95)**	**1.26 (1.003**–**1.59)**	1.03 (0.80–1.32)	0.93 (0.70–1.22)	0.85 (0.63–1.17)	1.11 (0.77–1.58)
PT, RT, or OT	**1.46 (1.13**–**1.88)**	1.13 (0.88–1.44)	1.13 (0.86–1.47)	0.90 (0.67–1.21)	0.79 (0.57–1.09)	0.97 (0.67–1.40)
SMT	**2.20 (1.49**–**3.25)**	1.08 (0.78–1.48)	0.99 (0.69–1.43)	1.27 (0.88–1.83)	1.17 (0.77–1.78)	1.01 (0.64–1.60)
Talk therapies	1.21 (0.71–2.06)	0.59 (0.36–0.94)	1.02 (0.59–1.74)	1.53 (0.94–2.49)	1.66 (0.99–2.78)	**1.80 (1.04**–**3.11)**
Self-management	**1.98 (1.28**–**3.06)**	0.75 (0.50–1.11)	1.20 (0.76–1.89)	1.38 (0.91–2.08)	0.94 (0.59–1.48)	1.08 (0.65–1.79)
Group/peer support	0.52 (0.27–1.02)	1.03 (0.48–2.23)	1.71 (0.78–3.73)	1.32 (0.69–2.51)	1.09 (0.57–2.09)	**2.24 (1.10**–**4.56)**
Yoga or Tai Chi	1.34 (0.85–2.11)	**1.67 (1.09**–**2.57)**	1.30 (0.82–2.06)	0.74 (0.45–1.21)	0.74 (0.42–1.30)	1.67 (0.98–2.85)
Massage	**1.51 (1.10**–**2.09)**	**1.55 (1.16**–**2.07)**	1.22 (0.88–1.69)	1.22 (0.89–1.69)	0.94 (0.65–1.37)	1.06 (0.69–1.63)
Meditation, GI, or other relaxation	1.11 (0.84–1.46)	1.21 (0.92–1.59)	1.21 (0.89–1.65)	0.79 (0.58–1.08)	1.37 (0.997–1.89)	**1.82 (1.26**–**2.61)**

*Note:* Each model adjusted for sex, age, race, marital status, income status, employment status, education status, urbanicity, body mass index, and number of medical conditions. Bolded results indicate statistical significance.

Abbreviations: CI, confidence interval; GI, guided imagery; NPI, nonpharmacologic intervention; OR, odds ratio; OT, occupational therapy; PT, physical therapy; RT, rehabilitative therapy; SMT, spinal manipulation therapy.

**Table 5:** Adjusted logistic regression estimates for NPI use outcomes, OR (95% CI), in relation to the number of pain sites for patients with high-impact chronic pain.

	Number of bothersome pain sites
NPI ≥ 1	**1.11 (1.01**–**1.21)**
PT, RT, or OT	1.08 (0.99–1.18)
SMT	**1.18 (1.04**–**1.33)**
Talk therapies	**1.29 (1.05**–**1.60)**
Self-management	1.10 (0.94–1.26)
Group/peer support	1.17 (0.89–1.55)
Yoga or Tai Chi	**1.24 (1.02**–**1.51)**
Massage	**1.29 (1.17**–**1.43)**
Meditation, GI, or other relaxation	**1.25 (1.12**–**1.39)**

*Note:* Each model adjusted for sex, age, race, marital status, income status, employment status, education status, urbanicity, body mass index, and number of medical conditions. Bolded results indicate statistical significance.

Abbreviations: CI, confidence interval; GI, guided imagery; NPI, nonpharmacologic intervention; OR, odds ratio; OT, occupational therapy; PT, physical therapy; RT, rehabilitative therapy; SMT, spinal manipulation therapy.

## Data Availability

The data that support the findings of this study are available in the National Center for Health Statistics at https://www.cdc.gov/rdc/index.html. These data were derived from the following resources available in the public domain: 2019 NHIS Questionnaires, Datasets, and Documentation, https://www.cdc.gov/nchs/nhis/documentation/2019-nhis.html.
